# Preliminary study of ultra-widefield peripheral retinal angiographic patterns in children and its association to the perinatal condition

**DOI:** 10.1038/s41598-020-70677-9

**Published:** 2020-08-12

**Authors:** Jin Young Kim, Mi Young Choi, Eoi Jong Seo, Seungheon Lee, Ji Soo Kim, Ju Byung Chae, Dong Yoon Kim, June-Gone Kim

**Affiliations:** 1 Department of Ophthalmology, Jeju National University Hospital, Jeju National University School of Medicine, Jeju, South Korea; 2 Department of Ophthalmology, Chungbuk National University Hospital, College of Medicine, Chungbuk National University, 776, Sunhwan-1-Ro, Seowon-Gu, Cheongju 28644 South Korea; 3 Department of Ophthalmology, Asan Medical Center, University of Ulsan College of Medicine, Seoul, South Korea

**Keywords:** Eye diseases, Angiogenesis

## Abstract

This preliminary study analyzed the peripheral retinal vascular pattern in children, using ultra-widefield fluorescein angiography, and its association with perinatal conditions. Retrospective review was conducted examining the fluorescein angiographic findings of children with amblyopia (January 2017 to December 2018). We categorized the peripheral vascular patterns into two groups: loop and branching patterns. We investigated differences in these patterns, according to the perinatal condition. Thirty children (9.27 ± 3.41 years old; 47.67% male) were included. An equal number of children had the loop or branching pattern (15:15). The gestational age (GA) in the loop group was significantly shorter than the branching group (32.92 ± 5.62 vs. 36.67 ± 5.63 weeks, *p* = 0.04). The birth weight of the loop group was significantly lower than the branching group (2.00 ± 1.03 vs. 2.72 ± 0.93 kg, *p* = 0.03). Gender, age, delivery-type, and visual acuity, were not different between the groups. Lower birth weight and shorter GA were observed in children with the loop pattern. The difference in peripheral retinal vascular patterns, according to birth weight and GA, might be due to the development of immature retinal vessels at birth.

## Introduction

With advances in ultra-widefield fluorescein angiography (FA), several reports about peripheral retinal fluorescein angiographic findings of retinal vascular diseases (e.g., retinal vein occlusion, uveitis, and diabetes) have been published^1–9^. The peripheral fluorescein angiographic findings of normal subjects have been also evaluated by several researchers^10–13^. They reported that even in normal subjects, several anatomic variations of peripheral angiographic findings have been observed. Shah et al. and Lu et al. reported normal variation of peripheral FA findings^10,12^. Furthermore, interestingly, Singer et al. recently reported that the distances from disc to the perfused vascular border were significantly shorter in older subjects (> 60 years) than in younger subjects^14^. This means peripheral angiographic findings could change with age.

Seo et al. reported that the peripheral vascular pattern of normal healthy subjects including two possible distinctive peripheral vascular patterns: loop or branching patterns^13^. The loop peripheral vascular pattern, where the far-most peripheral vessels run circumferentially, has been known to be frequently observed in hypoxic conditions such as retinopathy of premature (ROP) or sickle cell retinopathy^15^. If there is any local hypoxia at the peripheral retina due to immature vasculogenesis at birth, the increased vascular endothelial growth factor (VEGF) secretion could promote endothelial cell growth, which could contribute to making the farthest vessel. However, in the far-peripheral retina, near the ora serrata, the retina is thin enough to derive oxygen supply from choroidal circulation^16,17^. So, in the far-peripheral retina, there might be no drive for hypoxia-mediated VEGF-induced retinal vascular growth. Since the peripheral retina is supplied with oxygen through choroid circulation, blood vessels do not have to grow in that direction (anteriorly, in a branching pattern). Instead of having anterior growth of blood vessels, the direction of blood vessel growth might turn towards or stay where oxygenation is relatively poor. We think this is probably the reason why the circumferential vascular pattern (loop pattern) vessels are formed along the edge of the peripheral retina.

In this background, we speculated that the peripheral vascular pattern might be different according to vasculogenesis at birth. If further vasculogenesis is needed after birth, due to a short gestational age or low birth weight, we thought the possibility of having a loop peripheral vascular pattern might be increased. Therefore, we conducted a preliminary study about peripheral retinal vascular patterns in children, using ultra-widefield FA, and its association with the perinatal condition. The primary objective of this study was to analyze the peripheral retinal vascular pattern in children, using ultra-widefield FA, and to investigate its association with the perinatal condition. The secondary objective was to analyze peripheral angiographic findings of children (e.g., the presence of capillary telangiectasia, microaneurysm, and vascular leakage in the late phase).

## Methods

A retrospective review was conducted based on children with amblyopia who visited pediatric ophthalmologic clinic and underwent ultra-widefield FA at Chungbuk National University Hospital in Cheongju, South Korea, between January 2017 and December 2018. This study was approved by the Institutional Review Board of Chungbuk National University Hospital and followed the tenets of the Declaration of Helsinki (2019-04-006). And because of the retrospective study design, this research involved no more than minimal risk to the subjects. Therefore, the informed consent was waived by the Institutional Review Board of Chungbuk National University Hospital.

The inclusion criteria were: (1) children with amblyopia who underwent FA to find the cause of decreased visual acuity, and (2) agreed to do ultra-widefield FA. The exclusion criteria were: (1) children who had a history of ROP, (2) any abnormalities in fundus or spectral domain-optical coherence tomography (SD-OCT) examination, (3) patients who have vascular diseases which could affect angiographic findings, (4) children who cannot cooperate with FA examinations, (5) patients with high myopia (> 7 diopters), glaucoma, or media opacities caused by cataracts or corneal disease, vitreous hemorrhage, or comorbid retinal disease, and (6) those whose fluorescein angiographic images were of poor quality.

### Ophthalmic examination and perinatal conditions

When amblyopic children visited our pediatric ophthalmologic clinic, they underwent a comprehensive ophthalmic examination, including best-corrected visual acuity (BCVA), using the Snellen chart, refractive error assessment, pneumotonometry, slit-lamp examination, dilated fundus examination, color fundus photography, SD-OCT (Spectralis; Heidelberg Engineering, Heidelberg, Germany), and FA (HRA; Heidelberg Engineering, Heidelberg, Germany). Ultra-widefield FA was conducted to obtain images of the far-peripheral retina. After the eyes were dilated with tropicamide phenylephrine, all patients received an intravenous injection of 5 mL 10% sodium fluorescein through the antecubital vein. For the ultra-widefield fluorescein angiographic image, the primary field was centered on the fovea, and images of the peripheral retina were captured by instructing the patient to stare at the superotemporal, superonasal, inferotemporal, inferonasal side to the greatest extent possible. From medical record, we collected demographic data and information about the perinatal condition, including age, sex, gestational age (GA), birth weight, and the type of delivery.

### FA examination and interpretation

**Figure 1:**
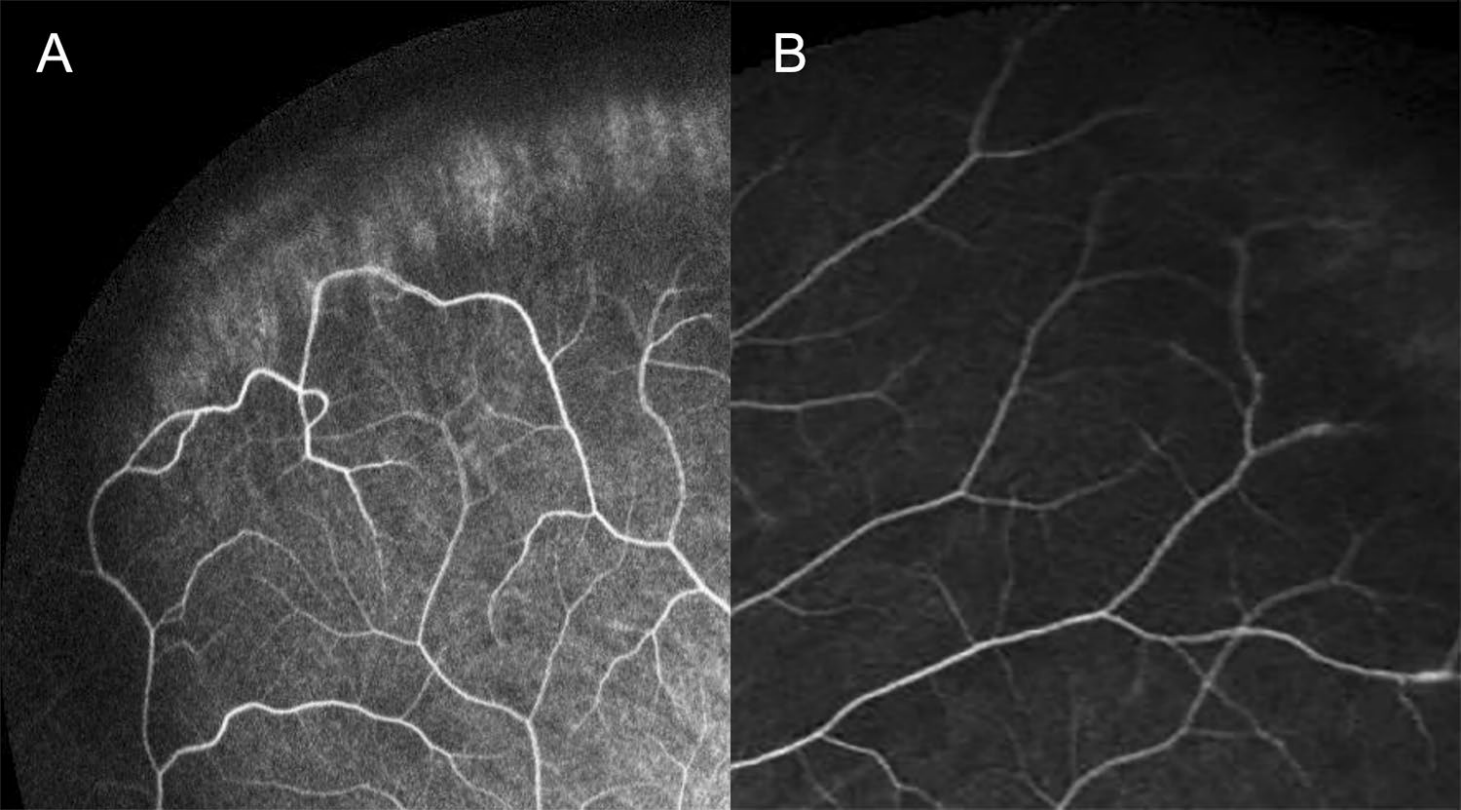
Representative images of ultra-widefield peripheral fluorescein angiographic patterns. Representative images of peripheral angiography. (**A**) Loop pattern peripheral angiographic finding. The most terminal vascular branches ran circumferentially and formed vascular loops. (**B**) Branching pattern peripheral angiographic finding. The most vascular endings were ramified and tapered toward the periphery.

The better seeing eye of the amblyopic patients was used for the ultra-widefield fluorescein angiographic image analysis. We divided each of the peripheral vascular patterns into groups based on the loop or branching pattern^13^. According to the previous study, we classified the pattern as “loop pattern” when most terminal vascular branches ran circumferentially and formed vascular loops. Conversely, when most vascular endings were ramified and tapered toward the periphery, we defined this pattern as “branching pattern”^13^. The type of peripheral vascular patterns was determined by 2 different independent retinal specialist (JYK and JBC) who were blinded to the study design and purpose. If there was any disagreement between two graders, another retinal specialist (DYK) finally determined peripheral vascular patterns. Representative images for the loop and branching pattern of peripheral angiography are shown in Fig. 1 (A: loop pattern, B: branching pattern). We also analyzed the images and documented the presence of capillary telangiectasia, microaneurysm, and vascular leakage in the late phase.

### Statistical analysis

The Statistical Package for the Social Sciences version 22.0 (SPSS, Inc., Chicago, IL, USA) was used for statistical analyses; *P* < 0.05 was considered statistically significant. To compare the perinatal condition, according to the peripheral angiographic findings, the Mann–Whitney U test or the Pearson chi-square test was used. Means of continuous variables were analyzed using the Mann–Whitney U test. Differences in the proportions between groups were tested using the Pearson chi-square test and the Fisher exact test.

### Meeting presentations

The 121st Annual Meeting of the Korean Ophthalmological Society, Busan, South Korea.

## Results

Table 1 represented the demographics of the included patients. Thirty children were included. The mean age of the children was 9.27 ± 3.41 years; there were 14 boys (47.67%). The mean GA at birth and the mean birth weights were 35.00 ± 5.38 weeks and 2.40 ± 1.02 kg, respectively.

**Table 1:** Demographics of included subjects.

Number of patients	30
Age (years)	9.27 ± 3.41
Sex (male/female)	14/16
Refractive error (S.E.)	− 0.89 ± 2.27
LogMAR BCVA	0.23 ± 0.20
Gestational age (weeks)	35.00 ± 5.38
Birth weight (kg)	2.40 ± 1.02

S.E., spherical equivalent; logMAR, logarithm of the minimal angle of resolution; BCVA, best-corrected visual acuity; kg, Kilogram.

### Peripheral angiographic findings and its association to the perinatal condition

**Figure 2:**
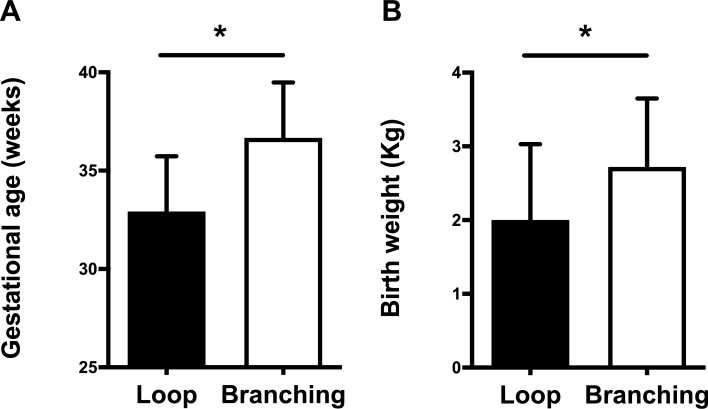
Peripheral angiographic findings and the perinatal condition. (**A**) The gestational age of the loop group was significantly shorter than the branching group (32.92 ± 5.62 vs. 36.67 ± 5.63 weeks, p = 0.04). (**B**) The birth weight of the loop was significantly lower than the branching group (2.00 ± 1.03 vs. 2.72 ± 0.93 kg, p = 0.03).

Among the included children, 15 children were fell into the loop pattern group (loop group) and the other 15 children fell into the branching pattern group (branching group). The vascular pattern of both eyes in each child was not different. Table 2 and Fig. 2 shows the demographic and perinatal conditions according to the peripheral vascular patterns. The GA of the loop group was significantly shorter than the branching group (32.92 ± 5.62 vs. 36.67 ± 5.63 weeks, *p* = 0.04). The birth weight of the loop group was also significantly lower than the branching group (2.00 ± 1.03 vs. 2.72 ± 0.93 kg, *p* = 0.03). However, gender, age, delivery type, and best-corrected visual acuity were not significantly different between the two groups.

**Table 2:** Perinatal conditions according to the peripheral angiographic findings.

	Loop pattern	Branching pattern	p value
Number of patients	15	15	
Age (years)	9.60 ± 3.11	8.93 ± 3.77	0.60*
Sex (male/female)	9/6	5/10	0.27^#^
Gestational age (week)	32.92 ± 5.62	36.67 ± 5.63	0.04*
Birth weight (kg)	2.00 ± 1.03	2.72 ± 0.93	0.03*
**Type of delivery**		–	0.46^#^
Vaginal	5 (33%)	8 (53%)	
Cesarean section	10 (67%)	7 (47%)	
LogMAR BCVA	0.20 ± 0.16	0.26 ± 0.23	0.430
Spherical equivalent	− 0.93 ± 2.47	− 0.62 ± 2.22	0.715

LogMAR, logarithm of the minimal angle of resolution; BCVA, best-corrected visual acuity; kg, Kilogram.

*Independent t test.

^#^Pearson chi square test.

### Microvascular abnormality according to the peripheral angiographic pattern

**Figure 3:**
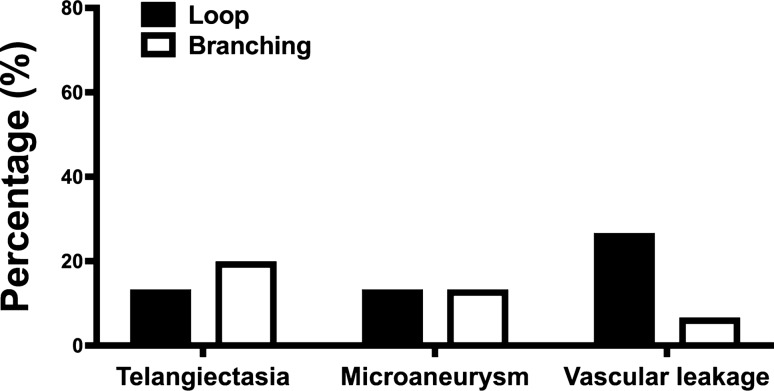
Microvascular abnormalities in children according to the peripheral angiographic pattern. The microvascular abnormalities such as telangiectasia, microaneurysm, and leakage were not different according to the peripheral angiographic pattern.

Figure 3 shows the microvascular abnormalities according to the peripheral angiographic pattern (loop vs. branching). The microvascular abnormalities in children were not different according to the peripheral angiographic pattern (telangiectasia: 13.3% vs. 20%, *p* = 0.624; microaneurysm: 13.3% vs. 13.3%, *p* = 1.000; vascular leakage: 26.7% vs. 6.7%, *p* = 0.330, in the loop and branching groups, respectively).

## Discussion

We analyzed the peripheral fluorescein angiographic findings of children and investigated their perinatal conditions, according to these findings. The primary observation of this study is that half of the children had a loop peripheral angiographic pattern. In children with this loop peripheral vascular pattern, their GA and birth weight were significantly shorter and lower, respectively, than those with a branching peripheral vascular pattern. Microvascular abnormalities in the children were not different according to their peripheral vascular patterns.

We previously reported peripheral angiographic findings of adults and found that there were two distinctive peripheral angiographic patterns: loop and branching^13^. The ratio of each peripheral vascular pattern in adults is nearly 50%. In the loop pattern, the most terminal vascular branches run circumferentially and form vascular loops. Conversely, in the branching pattern, most vascular endings are ramified and taper toward the periphery (Fig. 1).

The exact reason why there are two different peripheral angiographic patterns in healthy subjects is still unknown. In our previous report, we suggested the possible reason for why there are two different peripheral angiographic patterns. If peripheral vasculogenesis requires further progression after birth, circumferential vessels (the loop pattern peripheral angiogenesis) may form at the edge of the far peripheral retina, where the retina is thin enough to derive oxygen from the choroidal circulation^13,16^. The sufficient oxygen supply from the choroid circulation, at the far-peripheral retina, could decrease hypoxic-induced retinal angiogenesis. This may decrease the need for angiogenesis at that area and eventually lead to the loop peripheral angiographic pattern at the edge of far-peripheral retina^13,18,19^.

Based on this theory, we speculated that the peripheral angiographic pattern might be different due to the status of vasculogenesis at birth. In general, the normal vasculogenesis of the fetus is completed at 40 weeks of GA^20,21^. Therefore, if the infant is born earlier than 40 weeks of GA, the peripheral retinal vessel has to continue to develop after birth. Further angiogenesis of the peripheral retina should be needed with a lower GA. Hypoxic-induced VEGF secretion is the main driver for angiogenesis in the fetus^18–21^. However, in the far-peripheral retina, the need for vasculogenesis might be decreased because of the sufficient oxygen supply from choroidal circulation, which could contribute to the circumscribed pattern of the peripheral angiogenesis (loop pattern) rather than the branching pattern. Therefore, if further angiogenesis is needed, the probability of showing loop pattern angiographic findings might be increased. Thus, we speculated that in newborns with short GA or low birth weight, there is a higher chance of developing a loop vascular pattern.

In this study, children with loop peripheral angiographic patterns exhibited short GA and low birth weight. This result is consistent with our hypothesis that children whose peripheral retinal vessels required further development after birth are more likely to have a loop pattern peripheral angiographic finding. And we speculate that the vessel growth pattern of ROP might be an aggressive form of loop pattern which was observed in our study. Although we found that the peripheral blood vessel pattern may be different according to the GA, we could not determine the long-term effect of the vascular pattern on the development of certain vascular abnormalities in the future.

We found that microvascular abnormalities, according to the peripheral angiographic pattern in children, were not significantly different. However, we previously observed that microvascular abnormalities such as microaneurysm and vascular leakage were significantly more common in adults who had the loop peripheral vascular pattern^13^. We could not determine why only adults, rather than children, with loop peripheral vascular patterns had more microvascular abnormalities. We suggested a possible explanation for why adults with loop peripheral vascular patterns had more microvascular abnormalities in the previous report^13^. After the loop pattern is formed in childhood, relative hypo-oxygenation may have occurred due to further growth and thickening of the peripheral retina, decreasing oxygen supply from the choroid circulation. This relative hypo-oxygenation might increase the VEGF, which may eventually cause subsequent microvascular abnormalities (e.g., microaneurysm, telangiectasia, and vascular leakages). Therefore, considering more microvascular abnormalities were observed in adults rather than children, further research should be conducted to determine how each peripheral vascular pattern affects vascular abnormalities in the future.

A strength of the current study is that it provides a possible explanation for why some patients exhibited a loop peripheral vascular pattern. We explained that subjects with short GA and low birth weight showed a higher probability of exhibiting the loop peripheral vascular pattern. However, the present study also had some notable following limitations inherent in its retrospective design. (1) Though we excluded children who had a history of ROP, it is possible that some children with ROP were included as not all included children underwent an ROP screening examination. However, by excluding patients with any abnormal fundus or SD-OCT finding, we could minimize this bias. (2) The included amblyopic children cannot be assumed to be healthy subjects. However, for ethical reasons, we could not get the peripheral angiographic images of children without ocular problems. If there were no abnormal findings in the fundus or SD-OCT examinations, the children may be considered appropriate as a substitute for a healthy subject. (3) Finally, considering a small number included children and borderline p-value between the loop and branching vascular pattern, further study with large number of children should be needed to verify our study result. Despite these limitations, this study had a unique objective to analyze the peripheral retinal vascular patterns of children, using ultra-widefield FA, and to investigate the association with the perinatal conditions.

In conclusion, this preliminary study about peripheral vascular patterns according to the perinatal conditions showed that children with short GA and low birth weight more often exhibited a loop peripheral retina angiographic pattern. The ratio of each peripheral vascular pattern is equal in children. The difference in peripheral retinal vascular patterns, according to birth weight and GA, is thought to be due to the difference in the developmental status of immature retinal vessels at birth. In eyes with loop peripheral angiographic patterns, more microvascular abnormalities had developed with age. Therefore, further longitudinal studies with a large number of patients about the effect of the peripheral vascular patterns on vascular abnormalities are needed.
